# Increased gait variability during robot-assisted walking is accompanied by increased sensorimotor brain activity in healthy people

**DOI:** 10.1186/s12984-019-0636-3

**Published:** 2019-12-27

**Authors:** Alisa Berger, Fabian Horst, Fabian Steinberg, Fabian Thomas, Claudia Müller-Eising, Wolfgang I. Schöllhorn, Michael Doppelmayr

**Affiliations:** 1 Department of Sport Psychology, Institute of Sport Science, Johannes Gutenberg-University Mainz, Albert Schweitzer Straße 22, 55128 Mainz, Germany; 2 Department of Training and Movement Science, Institute of Sport Science, Johannes Gutenberg-University Mainz, Mainz, Germany; 3 School of Kinesiology, Louisiana State University, Baton Rouge, USA; 4 Center of Neurorehabilitation neuroneum, Bad Homburg, Germany; 5 Centre for Cognitive Neuroscience, Paris Lodron University of Salzburg, Salzburg, Austria

**Keywords:** Walking, Gait variability, GRF, Brain activity, Neuroimaging, Functional near-infrared spectroscopy, fNIRS, Robotic rehabilitation, RAGT, Neurorehabilitation

## Abstract

**Background:**

Gait disorders are major symptoms of neurological diseases affecting the quality of life. Interventions that restore walking and allow patients to maintain safe and independent mobility are essential. Robot-assisted gait training (RAGT) proved to be a promising treatment for restoring and improving the ability to walk. Due to heterogenuous study designs and fragmentary knowlegde about the neural correlates associated with RAGT and the relation to motor recovery, guidelines for an individually optimized therapy can hardly be derived. To optimize robotic rehabilitation, it is crucial to understand how robotic assistance affect locomotor control and its underlying brain activity. Thus, this study aimed to investigate the effects of robotic assistance (RA) during treadmill walking (TW) on cortical activity and the relationship between RA-related changes of cortical activity and biomechanical gait characteristics.

**Methods:**

Twelve healthy, right-handed volunteers (9 females; M = 25 ± 4 years) performed unassisted walking (UAW) and robot-assisted walking (RAW) trials on a treadmill, at 2.8 km/h, in a randomized, within-subject design. Ground reaction forces (GRFs) provided information regarding the individual gait patterns, while brain activity was examined by measuring cerebral hemodynamic changes in brain regions associated with the cortical locomotor network, including the sensorimotor cortex (SMC), premotor cortex (PMC) and supplementary motor area (SMA), using functional near-infrared spectroscopy (fNIRS).

**Results:**

A statistically significant increase in brain activity was observed in the SMC compared with the PMC and SMA (*p* < 0.05), and a classical double bump in the vertical GRF was observed during both UAW and RAW throughout the stance phase. However, intraindividual gait variability increased significantly with RA and was correlated with increased brain activity in the SMC (*p* = 0.05; *r* = 0.57).

**Conclusions:**

On the one hand, robotic guidance could generate sensory feedback that promotes active participation, leading to increased gait variability and somatosensory brain activity. On the other hand, changes in brain activity and biomechanical gait characteristics may also be due to the sensory feedback of the robot, which disrupts the cortical network of automated walking in healthy individuals. More comprehensive neurophysiological studies both in laboratory and in clinical settings are necessary to investigate the entire brain network associated with RAW.

## Background

Safe and independent locomotion represents a fundamental motor function for humans that is essential for self-contained living and good quality of life [1–5]. Locomotion requires the ability to coordinate a number of different muscles acting on different joints [6–8], which are guided by cortical and subcortical brain structures within the locomotor network [9]. Structural and functional changes within the locomotor network are often accompanied by gait and balance impairments which are frequently considered to be the most significant concerns in individuals suffering from brain injuries or neurological diseases [5, 10, 11]. Reduced walking speeds and step lengths [12] as well as non-optimal amount of gait variability [13–15] are common symptoms associated with gait impairments that increase the risk of falling [16].

In addition to manual-assisted therapy, robotic neurorehabilitation has often been applied in recent years [17, 18] because it provides early, intensive, task-specific and multi-sensory training which is thought to be effective for balance and gait recovery [17, 19, 20]. Depending on the severity of the disease, movements can be completely guided or assisted, tailored to individual needs [17], using either stationary robotic systems or wearable powered exoskeletons.

Previous studies investigated the effectiveness of robot-assisted gait training (RAGT) in patients suffering from stroke [21, 22], multiple sclerosis [23–26], Parkinson’s disease [27, 28], traumatic brain injury [29] or spinal cord injury [30–32]. Positive effects of RAGT on walking speed [33, 34], leg muscle force [23] step length, and gait symmetry [29, 35] were reported. However, the results of different studies are difficult to summarize due to the lack of consistency in protocols and settings of robotic-assisted treatments (e.g., amount and frequency of training sessions, amount and type of provided robotic support) as well as fragmentary knowledge of the effects on functional brain reorganization, motor recovery and their relation [36, 37]. Therefore, it is currently a huge challenge to draw guidelines for robotic rehabilitation protocols [22, 36–38]. To design prologned personalized training protocols in robotic rehabilitation to maximize individual treatment effects [37], it is crucial to increase the understanding of changes in locomotor patterns [39] and brain signals [40] underlying RAGT and how they are related [36, 41].

A series of studies investigated the effects of robotic assistance (RA) on biomechanical gait patterns in healthy people [39, 42–44]. On one side, altered gait patterns were reported during robot-assisted walking (RAW) compared to unassisted walking (UAW), in particular, substantially higher muscle activity in the quadriceps, gluteus and adductor longus leg muscles and lower muscle activity in the gastrocnemius and tibialis anterior ankle muscles [39, 42] as well as reduced  lower-body joint angles due to the little medial-lateral hip movements [45–47]. On the other side, similar muscle activation patterns were observed during RAW compared to UAW [44, 48, 49], indicating that robotic devices allow physiological muscle activation patterns during gait [48]. However, it is hypothesized that the ability to execute a physiological gait pattern depends on how the training parameters such as body weight support (BWS), guidance force (GF) or kinematic restrictions in the robotic devices are set [44, 48, 50]. For example, Aurich-Schuler et al. [48] reported that the movements of the trunk and pelvis are more similar to UAW on a treadmill when the pelvis is not fixed during RAW, indicating that differences in musle activity and kinematic gait characteristics between RAW and UAW are due to the reduction in degrees of freedom that user’s experience while walking in the robotic device [45]. In line with this, a clinical concern that is often raised with respect to RAW is the lack of gait variability [45, 48, 50]. It is assumed that since the robotic systems are often operated with 100% GF, which means that the devices attempt to force a particular gait pattern regardless of the user’s intentions, the user lacks the ability to vary and adapt his gait patterns [45]. Contrary to this, Hidler et al. [45] observed differences in kinematic gait patterns between subsequent steps during RAW, as demonstrated by variability in relative knee and hip movements. Nevertheless, Gizzi et al. [49] showed that the muscular activity during RAW was clearly more stereotyped and similar among individuals compared to UAW. They concluded that RAW provides a therapeutic approach to restore and improve walking that is more repeatable and standardized than approaches based on exercising during UAW [49].

In addition to biomechanical gait changes, insights into brain activity and intervention-related changes in brain activity that relate to gait responses, will contribute to the optimization of therapy interventions [41, 51]. Whereas the application of functional magnetic resonance imaging (fMRI), considered as gold standard for the assessment of activity in cortical and subcortical structures, is restricted due to the vulnerability for movement artifacts and the range of motion in the scanner [52], functional near infrared spectroscopy (fNIRS) is affordable and easily implementable in a portable system, less susceptible to motion artifacts, thus facilitation a wider range of application with special cohorts (e.g., children, patients) and in everyday environments (e.g., during a therapeutic session of RAW or UAW) [53, 54]. Although with lower resolution compared to fMRI [55], fNIRS also relies on the principle of neurovascular coupling and allows the indirect evaluation of cortical activation [56, 57] based on hemodynamic changes which are analogous to the blood-oxygenation-level-dependent responses measured by fMRI [56]. Despite limited depth sensitivity, which restricts the measurement of brain activity to cortical layers, it is a promising tool to investigate the contribution of cortical areas to the neuromotor control of gross motor skills, such as walking [53]. Regarding the cortical correlates of walking, numerous studies identified either increaesed oxygenated hemoglobin (Hboxy) concentration changes in the sensorimotor cortex (SMC) by using fNIRS [53, 57–59] or suppressed alpha and beta power in sensorimotor areas by using electroencephalography (EEG) [60–62] demonstrating that motor cortex and corticospinal tract contribute directly to the muscle activity of locomotion [63]. However, brain activity during RAW [36, 61, 64–68], especially in patients [69, 70] or by using fNIRS [68, 69], is rarely studied [71].

Analyzing the effects of RA on brain activity in healthy volunteers, Knaepen et al. [36] reported significantly suppressed alpha and beta rhythms in the right sensory cortex during UAW compared to RAW with 100% GF and 0% BWS. Thus, significantly larger involvement of the SMC during UAW compared to RAW were concluded [36]. In contrast, increases of Hboxy were observed in motor areas during RAW compared UAW, leading to the conclusion that RA facilitated increased cortical activation within locomotor control systems [68]. Furthermore, Simis et al. [69] demonstrated the feasibility of fNIRS to evaluate the real-time activation of the primary motor cortex (M1) in both hemispheres during RAW in patients suffering from spinal cord injury. Two out of three patients exhibited enhanced M1 activation during RAW compared with standing which indicate the enhanced involvement of motor cortical areas in walking with RA [69].

To summarize, previous studies mostly focused the effects of RA on either gait characteristics or brain activity. Combined measurements investigating the effects of RA on both biomechanical and hemodynamic patterns might help for a better understanding of the neurophysiological mechanisms underlying gait and gait disorders as well as the effectiveness of robotic rehabilitation on motor recovery [37, 71]. Up to now, no consensus exists regarding how robotic devices should be designed, controlled or adjusted (i.e., device settings, such as the level of support) for synergistic interactions with the human body to achieve optimal neurorehabilitation [37, 72]. Therefore, further research concerning behavioral and neurophysiological mechanisms underlying RAW as well as the modulatory effect of RAGT on neuroplasticy and gait recovery are required giving the fact that such knowledge is of clinical relevance for the development of gait rehabilitation strategies.

Consequently, the central purpose of this study was to investigate both gait characteristics and hemodynamic activity during RAW to identify RAW-related changes in brain activity and their relationship to gait responses. Assuming that sensorimotor areas play a pivotal role within the cortical network of automatic gait [9, 53] and that RA affects gait and brain patterns in young, healthy volunteers [39, 42, 45, 68], we hypothesized that RA result in both altered gait and brain activity patterns. Based on previous studies, more stereotypical gait characteristics with less inter- and intraindividual variability are expected during RAW due to 100% GF and the fixed pelvis compared to UAW [45, 48], wheares brain activity in SMC can be either decreased [36] or increased [68].

## Methods

This study was performed in accordance with the Declaration of Helsinki. Experimental procedures were performed in accordance with the recommendations of the Deutsche Gesellschaft für Psychologie and were approved by the ethical committee of the Medical Association Hessen in Frankfurt (Germany). The participants were informed about all relevant study-related contents and gave their written consent prior to the initiation of the experiment.

### Participants

Twelve healthy subjects (9 female, 3 male; aged 25 ± 4 years), without any gait pathologies and free of extremity injuries, were recruited to participate in this study. All participants were right-handed, according to the Edinburg handedness-scale [73], without any neurological or psychological disorders and with normal or corrected-to-normal vision. All participants were requested to disclose pre-existing neurological and psychological conditions, medical conditions, drug intake, and alcohol or caffeine intake during the preceding week.

### Experimental equipment

**Fig. 1:**
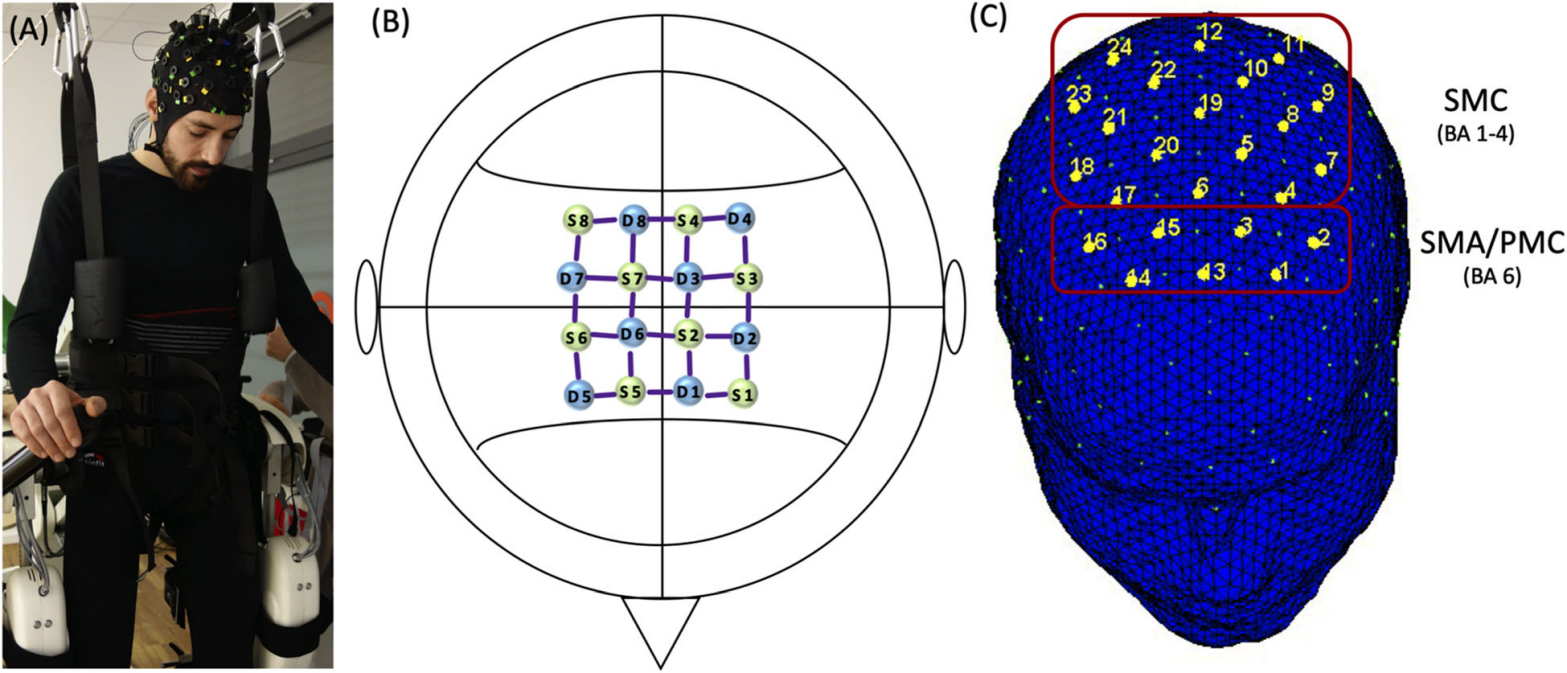
Montage and Setup. **a** Participant during robot-assisted walking (RAW), with functional near-infrared spectroscopy (fNIRS) montage. **b** fNIRS montage; S = Sources; D = Detectors **c** Classification of regions of interest (ROI): supplementary motor area/premotor cortex  (SMA/PMC) and sensorimotor cortex (SMC)

The Lokomat (Hocoma AG, Volketswil, Switzerland) is a robotic gait-orthosis, consisting of a motorized treadmill and a BWS system. Two robotic actuators can guide the knee and hip joints of participants to match pre-programmed gait patterns, which were derived from average joint trajectories of healthy walkers, using a GF ranging from 0 to 100% [74, 75] (Fig. 1a). Kinematic trajectories can be adjusted to each individual’s size and step preferences [45]. The BWS was adjusted to 30% body weight for each participant, and the control mode was set to provide 100% guidance [64].

Functional activation of the human cerebral cortex was recorded using a near-infrared optical tomographic imaging device (NIRSport, NIRx, Germany; Wavelengths: 760 nm, 850 nm; Sampling rate: 7.81 Hz). The methodology and the underlying physiology are explained in detail elsewhere [76]. A total of 16 optodes (8 emittors, 8 detectors) were placed with an interoptode distance of 3 cm [53, 54] above the motor cortex, based on the landmarks from the international 10–5 EEG system [77], resulting in 24 channels (source-detector pairs) of measurement (Fig. 1b). The spatial reolution was up to 1 cm. Head dimensions were individually measured and corresponding cap sizes assigned. Channel positions covered identical regions of both hemispheres including the SMC (Brodmann Area [BA] 1–4), and the supplementary motor area/premotor cortex  (SMA/PMC; BA6) (Fig. 1c).

Participants were equipped with standardized running shoes (Saucony Ride 9, Saucony, USA). Pressure insoles (Pedar mobile system, Novel GmbH, Germany) were inserted into the shoes for the synchronized measurement of plantar foot pressure, at a frequency of 100 Hz. Each insole consists of 99 capacitive sensors and covers the entire plantar area. The data recording process was managed by the software Novel Pedar-X Recorder 25.6.3 (Novel GmbH, Germany), and the vertical ground reaction force (GRF) was estimated for the analysis of kinetic and temporal gait variables.

### Experimental design

**Fig. 2:**
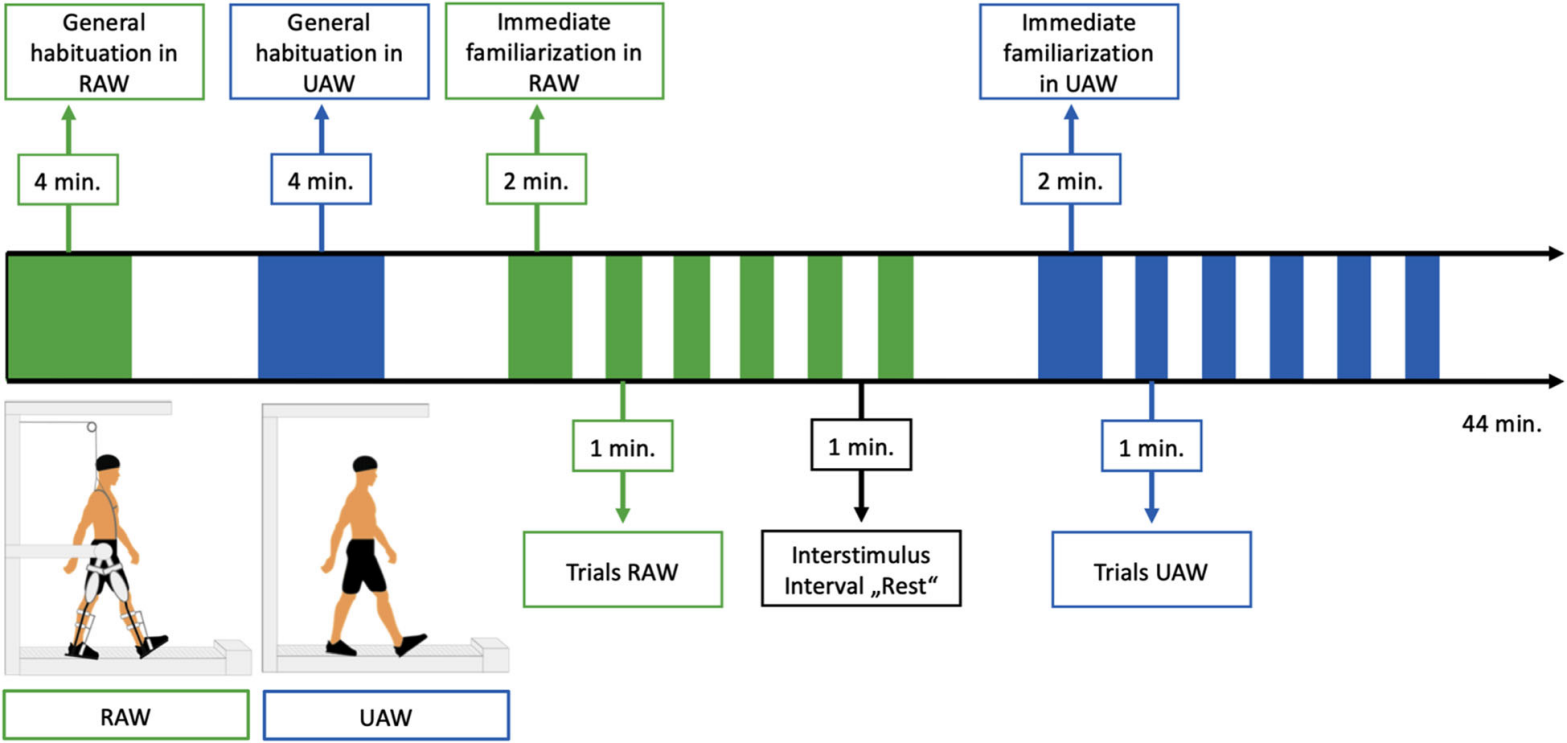
Study design and schematic illustration of unassisted walking (UAW) and robot-assisted walking (RAW)

Participants performed two blocks, (1) UAW and (2) RAW, in a randomized order. Each block consisted of five walking trials (60 s) and intertrail standing intervals of 60 s (s) [41, 53, 68, 78] (Fig. 2). While walking, the participants were instructed to actively follow the orthosis’s guidance while watching a neutral symbol (black cross) on a screen at eye level to ensure the most natural walking possible in an upright posture. During standing (rest), participants were instructed to stand with their feet shoulder-width apart while watching the same black cross. Furthermore, the participants were requested to avoid head movements and talking during the entire experiment, to reduce motion and physiological artifacts [78]. Prior to the experiment, individual adjustments of the Lokomat were undertaken, according to common practices in clinical therapy. The safety procedures of the rehabilitation center required that all subjects wore straps around the front foot to assist with ankle dorsiflexion. To familiarize themselves with the robotic device and treadmill walking (TW), participants walked with and without the Lokomat for 4 min before the experiment started.

### Data processing and analysis

fNIRS raw data were preprocessed and analyzed using the time series analysis routine available in the MATLAB-based NIRSlab analysis package (v2017.05, Nirx Medical Technologies, Glen Head, NY, [“Biomedical Optics”]) [79] following current recommendations when possible [53, 78]. In each channel of individual participant, fNIRS signal was visually inspected with respect to transient spikes and abrupt discontinuities which represent two most common forms of movement artifacts in fNIRS data. First, sections containing discontinuities (or “jumps”) as well as long term drifts were detected and corrected (standard deviation threshold = 5) [79]. Second, spikes were smoothed by a procedure that replaces contaminated data with the nearest signal [79]. Third, a band-pass filter (0.01 to 0.2 Hz) was applied to attenuate slow drifts and high frequency noises to reduce unknown global trend due to breathing, respiratory or cardiac rhythms, vasomotion, or other movement artifacts [59]. Then, time series of hemodynamic states of Hboxy and deoxygenated hemoglobin (Hbdeoxy) were computed using the the modified Beer-Lambert law [80, 81]. Following parameters were specified: wavelengths (WL1 = 760 nm; WL2 = 850 nm), differential pathlength factors (7.25 for WL1; 6.38 for WL2), interoptode distances (3 cm), background tissue values (totHb: 75 uM; MVO2Sat: 70%).

Preprocessed Hboxy concentration changes (∆Hboxy) were exported and processed as follows: 50 s per walking trial were used to analyze the hemodynamic responses during (1) UAW and (2) RAW due to the time needed for the acceleration and deceleration of the treadmill. The averaged baseline concentration values of rest before each walking trial were subtracted from the task-evoked concentration measurements, to account for time-dependent changes in cerebral oxygenation [78]. ∆Hboxy were calculated for regions of interest (ROI) (see Fig. 1c) during both UAW and RAW and used as a marker for the regional cortical activation, since it is more sensitive to locomotion-related activities than Hbdeoxy [82] and represents an accurate indicator of hemodynamic activity [83].

GRFs were preprocessed and analyzed using Matlab 2017b (MathWorks, USA). GRFs were filtered using a second-order Butterworth bidirectional low-pass filter, at a cut off frequency of 30 Hz. Offline processing included kinetic and temporal variables that were calculated based on stance-phase detection, using a GRF threshold of 50 N. The first and last ten stance phases (steps) from each of the five walking trials were excluded from the analysis because they corresponded with the acceleration and deceleration phases of the treadmill. The swing and stance phase times were measured. The stance phase was also subdivided into initial double-limb, single-limb and terminal double-limb support times. Furthermore, the number of steps and the cadence was calculated. Kinetic variables were analyzed during the stance phase of walking. The GRF values were normalized against body mass and were time-normalized against 101 data points corresponding with the stance phase of walking. Gait variability was estimated for time-continuous GRF during the stance phase, using the coefficient of variation (CV) [84]. According to Eq. (1), the intraindividual CV was calculated based on the mean ($\overline{GRF_{s,b,i}}$) and standard deviation (*σ*_*s*, *b*, *i*_) of the normalized GRF at the *i*-th interval of a concanated vector of the right and left leg stance phases. The intraindividual CV was calculated for each subject *s* and both blocks *b* (RAW and UAW)*.*

1 $$ IntraindividualCV\left(s,b\right)=\frac{\sqrt{\frac{1}{202}\ast {\sum}_{i=1}^{202}{\sigma_{s,b,i}}^2}}{\frac{1}{202}\ast {\sum}_{i=1}^{202}\mid \overline{GR{F}_{s,b,i}}\mid}\ast 100\left[\%\right] $$

Similarly, interindividual variability was estimated across the subject’s mean GRF, calculated across the time-continuous GRF from all stance phases from one subject. According to Eq. (2), the interindividual CV was calculated based on the mean ($\overline{GRF_{\overline{s},b,i}}$) and standard deviation (${\sigma}_{\overline{s},b,i}$) of the normalized subject’s mean GRF at the *i*-th interval of the concanated vector of the right and left leg stance phases. Interindividual CV was calculated for both blocks *b* (RAW and UAW)*.*

2 $$ InterindividualCV(b)=\frac{\sqrt{\frac{1}{202}\ast {\sum}_{i=1}^{202}{\sigma_{\overline{s},b,i}}^2}}{\frac{1}{202}\ast {\sum}_{i=1}^{202}\mid \overline{GR{F}_{\overline{s},b,i}}\mid}\ast 100\left[\%\right] $$

The absolute magnitude of the symmetry index, according to Herzog et al. [85], was adapted for *i* time-intervals of time-continuous GRF. The symmetry index (SI) is a method of assessing the differences between the variables associated with both lower limbs during walking. According to Eq. (3), the SI was calculated based on the absolute difference of the mean normalized GRF ($\overline{GRF\_{right}_i}$ and $\overline{GRF\_{left}_i}$) at the *i*-th interval for each subject *s* and both blocks *b* (RAW and UAW). An SI value of 0% indicates full symmetry, while an SI value > 0% indicates the degree of asymmetry [85].

3 $$ SI\left(s,b\right)=\frac{1}{101}\ast \left(\sum \limits_{i=1}^{101}\frac{\mid \overline{GR{F_{right}}_{s,b,i}}-\overline{GR{F_{left}}_{s,b,i}}\mid }{\frac{1}{2}\ast \mid \overline{GR{F_{right}}_{s,b,i}}+\overline{GR{F_{left}}_{s,b,i}}\mid}\ast 100\right)\left[\%\right] $$

Based on the time-continuous vertical GRF waveforms, three time-discrete variables were derived within the stance phase: the magnitude of the first peak (weight acceptance), the valley (mid-stance) and the magnitude of the second peak (push-off), as well as their temporal appearances during the stance phase.

The statistical analysis was conducted using SPSS 23 (IBM, Armonk, New York, USA). Normal distribution was examined for both hemodynamic and kinetic/temporal variables using the Shapiro-Wilk test (*p* ≥ 0.05). Averaged Hboxy values were computed for each subject and ROI (SMA/PMC, SMC) during both UAW and RAW [53, 78] and were normalized (normHboxy) by dividing them by the corresponding signal amplitude for the whole experiment [41, 59]. A two-way analysis of variance (ANOVA), with the factors condition (UAW or RAW) and ROI (SMA/PMC, SMC), was used to analyze differences in cortical hemodynamic patterns. In cases of significant main effects, Bonferroni-adjusted post hoc analyses provided statistical information regarding the differences among the ROIs by condition. Temporal and kinetic gait variables were statistically tested for differences between the experimental conditions (UAW and RAW) using paired t-tests. The overall level of significance was set to *p* ≤ 0.05. Mauchly’s test was used to check for any violations of sphericity. If a violation of sphericity was detected (*p* < 0.05) and a Greenhouse-Geisser epsilon ε > 0.75 existed, the Huynh-Feldt corrected *p*-values were reported. Otherwise (epsilon ε < 0.75), a Greenhouse-Geisser correction was applied. Effect sizes were given in partial eta-squared (ƞp^2^) or interpreted, according to Cohen. The association between cortical activation and gait characteristics was explored using Pearson’s correlation coefficient.

## Results

### Cortical activity (Hboxy)

**Fig. 3:**
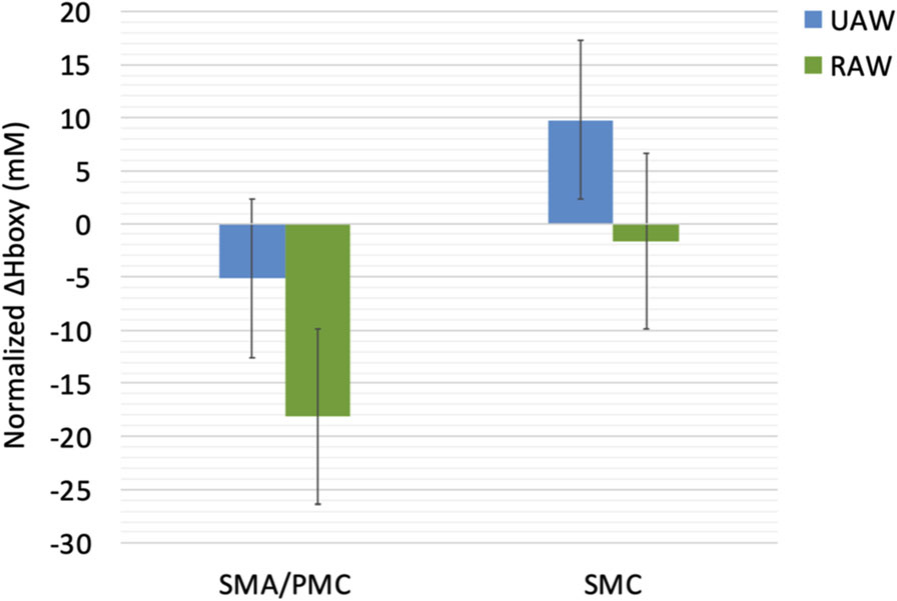
Normalized oxygenated hemoglobin (Hboxy; mean ± SME) for unassisted-walking (UAW) and robot-assisted walking (RAW). SMA/PMC, supplementary motor area/premotor cortex; SMC, sensorimotor cortex; SME = standard mean error

The effect of RAW on ∆Hboxy in locomotor cortical areas was analyzed using a two-way repeated measurements ANOVA with the factors ROI (SMA/PMC, SMC) and CONDITION (UAW, RAW). ∆Hboxy served as dependent variable. A significant main effect for ROI [F(1,11) = 11.610, *p* = 0.006, ƞp^2^ = 0.513] was found indicating significant greater ∆Hboxy values in the 7 channels (1–3,13–16) covering regions of the SMA/PMC [BA6] compared to the 17 channels (4–12 and 17–24) covering regions of the SMC [BA1–4] (*p* = 0.052), independent of the condition. Neither CONDITION [F(1,11) = 1.204, *p* = 0.296, ƞp^2^ = 0.099] nor the interaction ROI x CONDITION [F(1,11) = 0.092, *p* = 0.767, ƞp^2^ = 0.008] were significant (Fig. 3).

### Gait characteristics

**Fig. 4:**
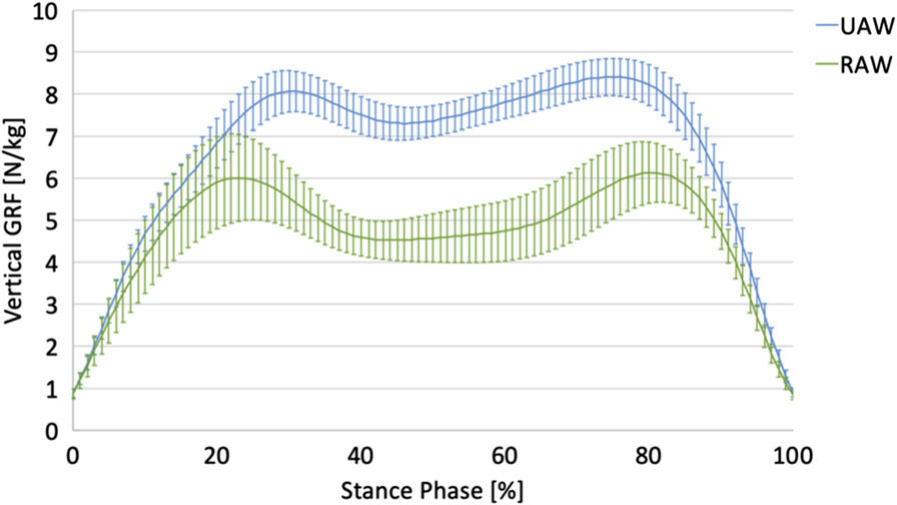
Normalized vertical ground reaction force (GRF; mean ± SD) during the stance phase of unassisted walking (UAW) and robot-assisted walking (RAW). In Additional file 1, normalized vertical GRF during the stance phase of UAW (Figure S1) and RAW (Figure S2) are presented  for each individual participant

Descriptive analyses of the mean vertical GRFs show a “classical” double bump (M-Shape) during the stance phase [84] for both UAW and RAW (Fig. 4). However, various differences in the gait characteristics were observed between the two conditions. First, the mean vertical GRFs were lower during RAW than during UAW. Second, the relative appearance of the peak values occurs earlier for the first peak and later for the second peak during RAW compared with UAW. Third, the vertical GRFs had higher standard deviations during RAW than during UAW. Statistical analyses of the time-discrete kinetic gait variables confirmed significantly lower GRFs and earlier and later appearances for the first and second vertical GRF peaks, respectively, during RAW than during UAW (Table 1)**.**

**Table 1:** Comparison of vertical ground reaction force variables (GRF; mean ± SD) during the stance phase of unassisted walking (UAW) and robot-assisted walking (RAW), SD = standard deviation

Kinetic Gait Variable	UAW	RAW	t	df	*p*	r
First Peak (weight acceptance) [N/kg]	8.2 ± 0.5	6.2 ± 1.0	6.807	11	0.000	0.899
First Peak (weight acceptance) [% Stance Phase]	31.4 ± 2.6	24.8 ± 3.2	7.366	11	0.000	0.912
Valley (mid-stance) [N/kg]	7.2 ± 0.4	4.2 ± 0.6	23.963	11	0.000	0.991
Valley (mid-stance) [% Stance Phase]	48.9 ± 2.9	49.6 ± 6.3	−0.277	11	0.787	0.083
Second Peak (weight acceptance) [N/kg]	8.6 ± 0.4	6.3 ± 0.7	12.898	11	0.000	0.968
Second Peak (weight acceptance) [% Stance Phase]	75.8 ± 3.0	82.4 ± 2.3	− 7.789	11	0.000	0.920
Intraindividual Variability [%]	5.8 ± 1.4	7.9 ± 1.3	− 4.427	11	0.001	0.800
Interindividual Variability [%]	7.0	15.7	–	–	–	–
Symmetry Index [%]	4.3 ± 2.0	7.7 ± 2.7	− 2.871	11	0.015	0.654

Fourth, significantly increased inter- and intraindividual variability and asymmetry between the time-continuous GRFs of the right and left feet (SI values) and significantly longer stance and swing phases emerge during RAW compared with UAW, despite the guidance of the robotic device and the same treadmill velocity (Table 2). Accordingly, lower numbers of steps and lower cadence values were observed during RAW than during UAW.

**Table 2:** Comparison of temporal gait variables (mean ± SD) during unassisted walking (UAW) and robot-assisted walking (RAW)

Temporal Gait Variable	UAW	RAW	t	df	*p*	r
Stance Phase [s]	0.84 ± 0.05	0.93 ± 0.03	5.78	11	0.000	0.867
Initial Double-Limb Support [s]	0.17 ± 0.02	0.19 ± 0.03	2.60	11	0.025	0.617
Single Limb Support [s]	0.50 ± 0.04	0.56 ± 0.03	4.82	11	0.001	0.824
Terminal Double-Limb Support [s]	0.17 ± 0.02	0.19 ± 0.03	2.78	11	0.018	0.643
Swing Phase [s]	0.50 ± 0.04	0.56 ± 0.03	4.89	11	0.000	0.828
Steps [number]	251.8 ± 24.4	189.1 ± 10.2	7.38	11	0.000	0.912
Cadence [steps/min]	91.1 ± 5.0	82.1 ± 0.1	6.03	11	0.000	0.876

### Association between changes in cortical activity and gait characteristics

**Fig. 5:**
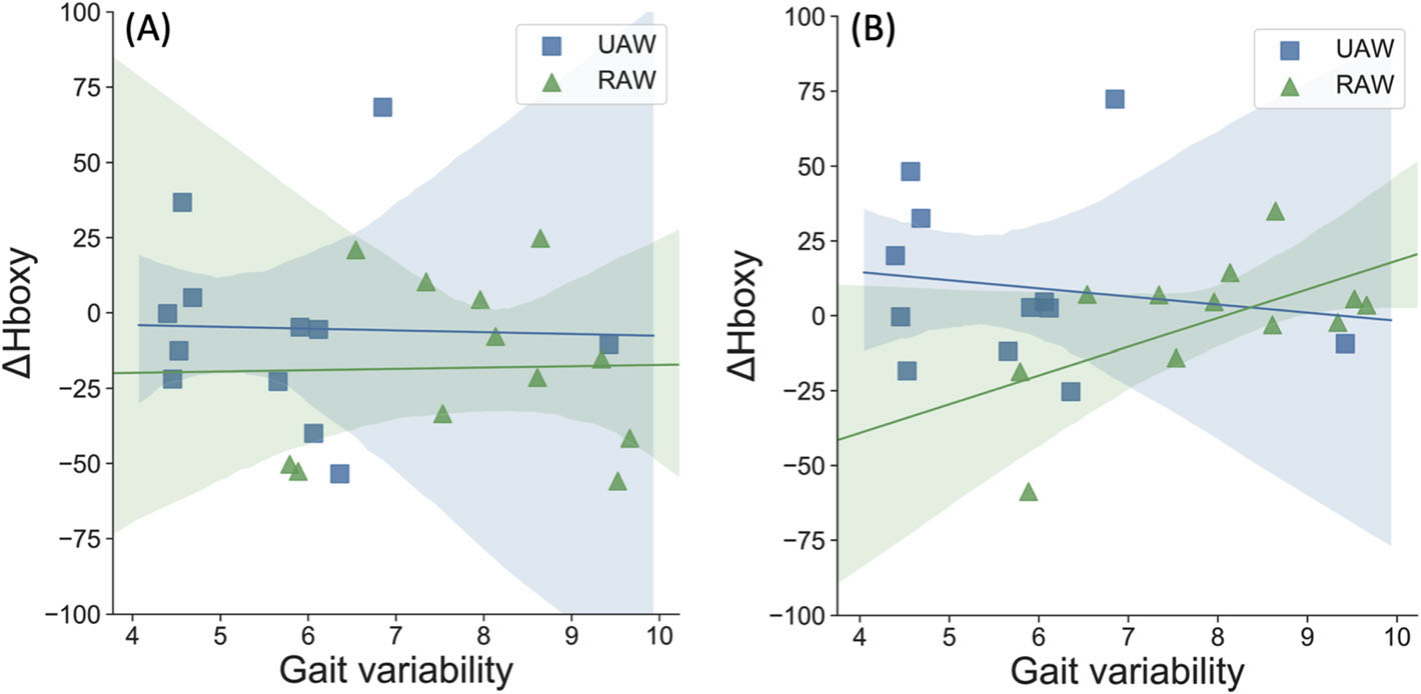
Correlations between relative oxygenated hemoglobin (Hboxy) and gait variability calculated by intraindividual coefficient of variation (CV) during unassisted-walking (UAW) and robot-assisted walking (RAW). **a** SMA/PMC, supplementary motor area/premotor cortex; **b** SMC, sensorimotor cortex; the shaded area represents the 95% confidence interval

Correlation analyses showed that changes in gait characteristics due to RA were also associated with changes in cortical activity. During RAW, a positive association between gait variability and Hboxy was observed only in the SMC (*p* = 0.052, *r* = 0.570). No further correlations were found during UAW or for other brain regions (SMA/PMC *p* = 0.951, *r* = 0.020). Thus, increased gait variability during RAW was associated with increased brain activity in the SMC (Fig. 5b).

## Discussion

In this study, the effects of RA on cortical activity during TW and the relationship to changes in gait characteristics were investigated. We identified a classical double bump in the GRF, throughout the stance phase during both UAW and RAW, which was accompanied by significantly increased brain activity in the SMC compared to premotor/supplementary motor areas. However, individual analyses showed significantly higher inter- and intraindividual gait variability due to RA that correlated with increased hemodynamic activity in the SMC (*p* = 0.052; *r* = 0.570).

In both conditions, shape characteristics of the mean GRF curves during the stance phase were observed. This in not in line with the results of Neckel et al. [46] who did not report a classical double bump during the stance phase during RAW, which could be due to the age differences of our samples. Furthermore, significantly altered kinematic patterns (lower GRF values and earlier and later appearances for the first and second vertical GRF peak values, respectively) as well as large inter- and intraindividual gait variability were observed during RAW compared to UAW. Results of the kinematic patterns are consistent with other biomechanical studies showing altered muscle activity [39, 42] or kinematic patterns [45–47] due to RA. The results of greater inter- and intraindividual gait variability during RAW do not agree with the more stereotypical and similar patterns of Gizzi et al. [49], nor with the assumption that the user lacks the ability to vary and adapt gait patterns during RAW [45, 48, 50].

Regarding brain activity during UAW, Hboxy concentration changes were significantly increased in sensorimotor areaes compared to areas of the SMA/PMC which is in line with other neurophysiological studies that showed increased Hboxy concentrations during walking [57, 58]. This is further confirmed by EEG studies reporting suppressed alpha and beta oscillations within the SMC [60–62] during active walking. This also demonstrates that the SMC and the corticospinal tract contribute directly to muscle activity in locomotion [9, 53, 63] representing a general marker of an active movemet-related neuronal state [61].

Analyzing the effects of RA on cortical patterns, significantly increased Hboxy concentration changes were also observed in SMC compared to frontal areas. Whereas Kim et al. [68] observed more global network activation during RAW compared to UAW, Knaepen et al. [36] reported significantly suppressed alpha and beta power during UAW compared to RAW with the conclusion that walking with 100% GF leads to less active participation and little activation of the SMC, which should be avoided during RAGT.

However, during RAW, we observed a positive correlation between ΔHboxy concentrations in the SMC and intraindividual gait variability. Thus, individuals with larger gait variability showed higher sensorimotor brain activity, which is similar to the results reported of Vitorio et al. [41]. In this study, positive correlations between gait variability and ΔHboxy in the PMC and M1 were found in young healthy adults when walking with rhythmic auditory cueing [41]. The following two possible explanations are suggested.

On one side, robotic guidance might induce additional and new sensory feedback that promotes active participation, resulting in high gait variability and increased brain activity. This possibility is supported by previous observations that muscles exhibited marked and structurally phased activity, even under full guidance conditions [39, 42, 86–88]. Van Kammen et al. [88] found muscle activity in the vastus lateralis, suggesting that the leg muscles are still activated during RAW as opposed to the muscles related to stability and propulsion, in which activity is reduced under guidance conditions. This finding is remarkable because, in this state, the exoskeleton is responsible for walking control, and theoretically, no voluntary activity from the performer is required [87, 89]. However, the instructions used in the present study (i.e., ‘actively move along with the device’) may have affected activity, as previous studies have shown that encouraging active involvement increases muscle activity [86, 87] as well as brain activity significantly during RAW [64]. More specifically, Wagner et al. [64] showed significantly suppressed alpha and beta power during active compared to passive RAW. Dobkin (1994) also showed that passive stepping can lead to task-specific sensory information that induces and modulates step-like electromyography activity [90]. Thus, high guidance might also promote active contribution. Particularly in patients who are not able to walk unassisted, successful stepping induces task-specific sensory information that may trigger plastic changes in the central nervous system [88, 91]. Since active participation and the production of variable movement patterns are prerequisites for activity-dependent neuroplasticity [7, 20, 89, 92–94], it is important to determine whether the activation of the SMC can be triggered by changes in the levels of GF, BWS and kinematic freedom in order to specifically provoke gait variability due to active participation of the patient [45, 48, 50]. High gait variability may indicate that people use multiple combinations of gait variables to walk more effectively [45, 95], resulting in better and faster improvements during robotic rehabilitation.

On other side, the sensory feedback from robot guidance could also disturb the brain network underlying automatic walking, leading to increased gait variability and sensorimotor activity. According to Vitorio et al. [41], the requirement to adapt to external stimuli leads to disturbances in automatic walking in young healthy people, resulting in higher gait variability and higher cortical costs. As previous study have shown, the ability to execute a physiological gait pattern depends on how the training parameters such as BWS, GF or kinematic freedom in the robotic devices are set. During RAW with fixed pelvis, significantly altered muscle activity [39, 42, 45] and kinematic patterns [48, 50] were found. In addition to GF, BWS and kinematic freedom, the presence of foot support may also contribute to altered patterns. The safety procedures of the therapy institution required that all subjects wear straps around the front foot to assist with ankle dorsiflexion, which is known to reduce activity in the ankle dorsiflexors [39, 42].

In summary, increased gait variability and sensorimotor activity during RAW could be the result of active participation or disrupted automatic locomotor control. However, the generalization of these results to other populations is not intended or recommended. Healthy elderly individuals [41] and patients with stroke [22], multiple sclerosis [23, 25, 26], Parkinson’s disease [27, 28], brain injuries [29] or spinal cord injuries [30, 31] who suffer from gait and balance disorders react differently to robotic support than healthy young people, which may lead to different gait and brain activation patterns [44]. In addition to high inter- and intraindividual variability within one sample, the heterogeneity of methodological procedures between studies appears to pose another challenge [71].

Therefore, one future goal should be to understand the mechanisms underlying RAGT and which parameters determine the effectiveness of a single treatment in the heterogenuous population of patients suffering from neurological diseases [37]. For this purpose, objective biomarkers for motor recovery and neuroplastic changes have to be identified [37]. Then, specific training protocols and further interventions, such as augmented feedback with virtual reality, brain-machine interface or non-invasive brain stimulation, can be developed to deliver sustainable therapies for individualized rehabilitation that optimizes the outcome and efficacy of gait recovery, which together can foster independent living and improve the quality of life for neurological patients [37, 71].

### Methodological limitations

Two methodological limitations that emerged using the present approach should be mentioned. First, the ability to walk is guided by an optimal interaction between cortical and subcortical brain structures within the locomotor network [53]. Using our NIRSport system, we were only able to report brain activity patterns in motor cortical areas and were unable to monitor the activities of subcortical areas or other cortical involvements. Various studies have reported that patients with gait disorders recruit additional cortical regions to manage the demands of UAW and RAW, due to structural and/or functional changes in the brain. Measuring the entire cortical network underlying locomotion may be necessary to investigate neuronal compensations and cognitive resources used for neuroplastic processes during gait rehabilitation. Therefore, we must be careful when discussing brain activity associated with other regions involved in locomotor control [9].

Secondly, we must take into account the small sample size of our healthy volunteers and their young age (mean: 25 ± 4 years), which also had no gait pathologies. Thus, RA guidance of gait movement might have different effects in elderly subjects or patients who are not able to walk without restrictions [96]. Therefore, the findings from our study are difficult to apply to other age or patient groups, as neurological patients often suffer from movement disorders and therefore use different control strategies during RAW. Although the available results provide relevant insights into the mobile applications of neurophysiological measurements during RAW, with approaches for further therapeutic interventions during robotic rehabilitation, the effects of RAW must also be investigated in other groups and in patients with gait disorders in the future.

## Conclusions

The purpose of the present study was to investigate brain activity during UAW and RAW and how this activity was associated with gait characteristics. The results confirmed the involvement of the SMC during TW and significantly increased gait variability due to RA, which correlated positively with brain activity. Furthermore, this study highlights the interaction between cortical activity and gait variability, stressing the need to use holistic, multisystem approaches when investigating TW in elderly individuals or patients suffering from gait disorders. Assessing the effects of RA on brain activity and gait characteristics is essential to develop a better understanding of how robotic devices affect human locomotion. This knowledge is essential for interventional studies examining the rehabilitation of motor disorders. Basic research regarding robotic rehabilitation is necessary to gain a deeper understanding of the brain and gait patterns associated with RAW, which is essential for further investigations of gait recovery and neuroplastic changes. In addition, clinical longitudinal studies are required to identify individual gait improvements and to identify the underlying neurophysiological changes to develop therapies with respect to interindividual differences. RAGT devices should be designed to provide an amount of force that adapts to the patient’s capacity, to achieve an optimal balance between forced motor activity and the promotion of the patient’s voluntary activity [36, 92–94]. Further combined studies are necessary to determine the relationship between brain activity and functional motor improvements and to evaluate the effects of therapeutic interventions. Neurophysiological investigations can contribute to the development of robotic rehabilitation and to individual, closed-loop treatments for future neurorehabilitation therapies.

## Supplementary information

**Additional file 1: Figure S1.** Normalized vertical ground reaction force (GRF; mean) during the stance phase of unassisted walking (UAW) for each individual participant. **Figure S2.** Normalized vertical ground reaction force (GRF; mean) during the stance phase of robot-assisted walking (RAW) for each individual participant.

## Data Availability

The datasets used and analysed during the current study are available from the corresponding author on reasonable request.

## Abbreviations

ANOVA: Analysis of variance
BA: Brodmann area
BWS: Body weight support
EEG: Electroencephalography
fNIRS: Functional nearinfrared spectroscopy
GF: Guidance force
GRF: Ground reaction forces
Hbdeoxy: Deoxygenated hemoglobin
Hboxy: Oxygenated hemoglobin
M1: Primary motor cortex
RA: Robotic assistance
RAGT: Robot assisted gait training
RAW: Robot assisted walking
ROI: Regions of interest
SD: Standard deviation
SEM: Standard mean error
SI: Symmetry index
SMA: Supplementary motor area
SMC: Sensorimotor cortex
TW: Treadmill walking
UAW: Unassisted walking
ΔHboxy: Relative changes of oxygenated hemoglobin
