# Supplementary material for: Increased gait variability during robot-assisted walking is accompanied by increased sensorimotor brain activity in healthy people
Source: J Neuroeng Rehabil. 2019 Dec 27;16:161. doi: 10.1186/s12984-019-0636-3 (PMC6935063; doi:10.1186/s12984-019-0636-3)
Supplement: Supplementary file 1 — Additional file 1: Figure S1. Normalized vertical ground reaction force (GRF; mean) during the stance phase of unassisted walking (UAW) for each individual participant. Figure S2. Normalized vertical ground reaction force (GRF; mean) during the stance phase of robot-assisted walking (RAW) for each individual participant. [file 12984_2019_636_MOESM1_ESM.pptx]

## Slide 1
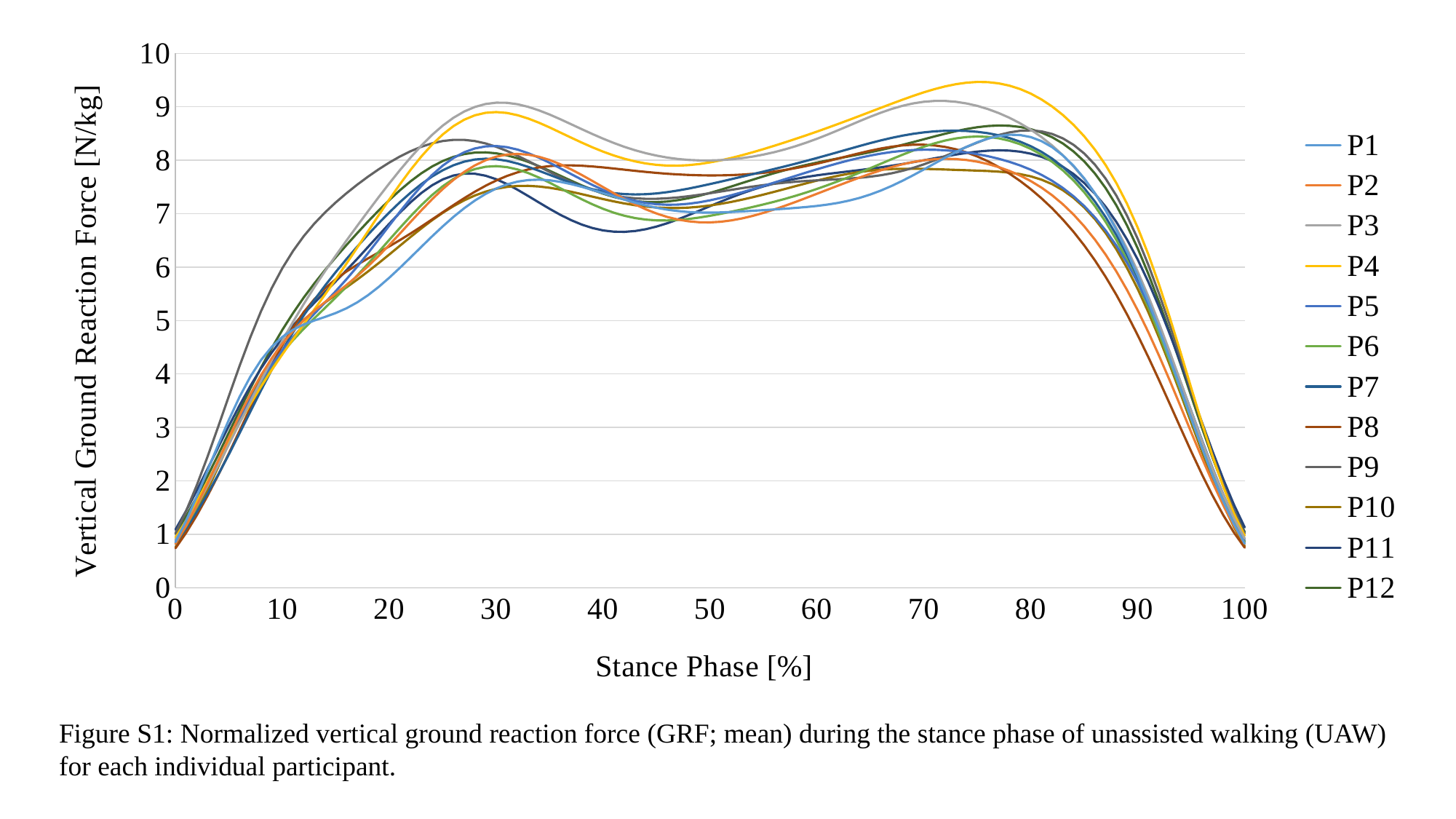

### Chart
| Category | P1 | P2 | P3 | P4 | P5 | P6 | P7 | P8 | P9 | P10 | P11 | P12 |
|---|---|---|---|---|---|---|---|---|---|---|---|---|Figure S1: Normalized vertical ground reaction force (GRF; mean) during the stance phase of unassisted walking (UAW) for each individual participant.

## Slide 2
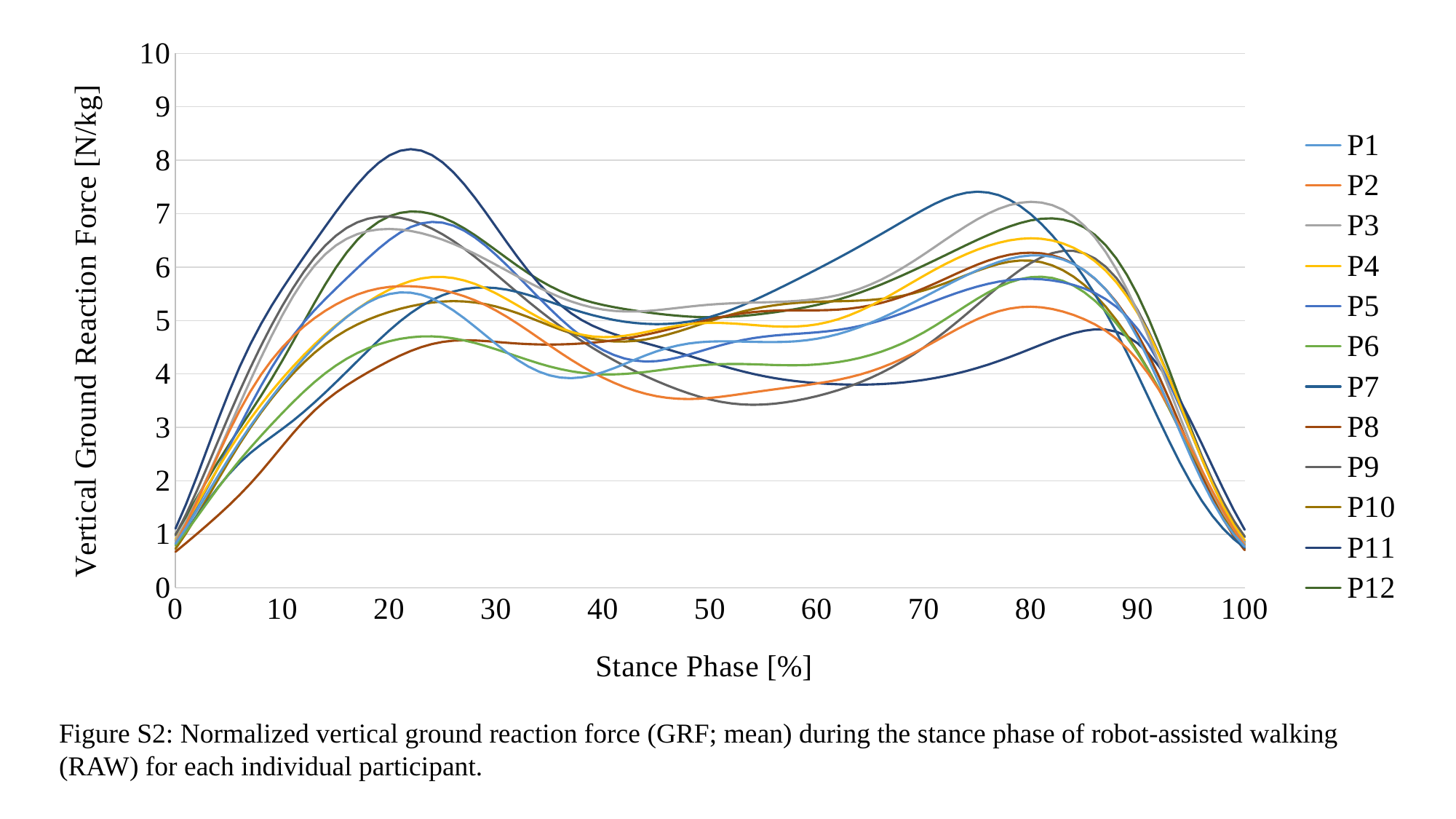

### Chart
| Category | P1 | P2 | P3 | P4 | P5 | P6 | P7 | P8 | P9 | P10 | P11 | P12 |
|---|---|---|---|---|---|---|---|---|---|---|---|---|Figure S2: Normalized vertical ground reaction force (GRF; mean) during the stance phase of robot-assisted walking (RAW) for each individual participant.
